# Saikosaponin D alleviates inflammatory response of osteoarthritis and mediates autophagy via elevating microRNA-199-3p to target transcription Factor-4

**DOI:** 10.1186/s13018-024-04607-0

**Published:** 2024-02-22

**Authors:** Ming Yan, DaWei Zhang, Min Yang

**Affiliations:** Department of Orthopedics, The First Affiliated Hospital of Air Force Military Medical University, No. 128, Changle West Road, Xincheng District, Xi’an City, 710000 Shaanxi Province China

**Keywords:** Osteoarthritis, Saikosaponin D, MicroRNA-42, Transcription Factor-4, Autophagy

## Abstract

**Objective:**

This study was to investigate the underlying mechanism by which Saikosaponin D (SSD) mitigates the inflammatory response associated with osteoarthritis (OA) and regulates autophagy through upregulation of microRNA (miR)-199-3p and downregulation of transcription Factor-4 (TCF4).

**Methods:**

A mouse OA model was established. Mice were intragastrically administered with SSD (0, 5, 10 μmol/L) or injected with miR-199-3p antagomir into the knee. Then, pathological changes in cartilage tissues were observed. Normal chondrocytes and OA chondrocytes were isolated and identified. Chondrocytes were treated with SSD and/or transfected with oligonucleotides or plasmid vectors targeting miR-199-3p and TCF4. Cell viability, apoptosis, inflammation, and autophagy were assessed. miR-199-3p and TCF4 expressions were measured, and their targeting relationship was analyzed.

**Results:**

In in vivo experiments, SSD ameliorated cartilage histopathological damage, decreased inflammatory factor content and promoted autophagy in OA mice. miR-199-3p expression was downregulated and TCF4 expression was upregulated in cartilage tissues of OA mice. miR-199-3p expression was upregulated and TCF4 expression was downregulated after SSD treatment. Downregulation of miR-199-3p attenuated the effect of SSD on OA mice. In in vitro experiments, SSD inhibited the inflammatory response and promoted autophagy in OA chondrocytes. Downregulation of miR-199-3p attenuated the effect of SSD on OA chondrocytes. In addition, upregulation of miR-199-3p alone inhibited inflammatory responses and promoted autophagy in OA chondrocytes. miR-199-3p targeted TCF4. Upregulation of TCF4 attenuated the effects of miR-199-3p upregulation on OA chondrocytes.

**Conclusions:**

SSD alleviates inflammatory response and mediates autophagy in OA via elevating miR-199-3p to target TCF4.

**Supplementary Information:**

The online version contains supplementary material available at 10.1186/s13018-024-04607-0.

## Introduction

Osteoarthritis (OA), a degenerative disease affecting the bones, presents clinically as joint pain, restricted movement, and stiffness. Its essential pathological characteristics include the degeneration of articular cartilage (AC), alterations in the subchondral bone, and inflammation of the surrounding soft tissues [1, 2]. OA demonstrates a significantly heightened prevalence. Specifically, the incidence of OA witnessed a substantial surge of 102% in 2017 when compared to the figures recorded in 1990 [3]. Joint replacement surgery (JRS) is widely recognized as the singular therapeutic intervention for advanced OA [4, 5]. Despite this, surgical treatment imposes a serious economic burden on patients and even society, not to mention the limitations of JRS, including persistent pain and limited prostheses [6]. Joint replacement surgery (JRS) is widely acknowledged as the singular remedy for advanced OA, thus necessitating the exploration of therapeutic targets for OA [5].

Numerous studies have speculated that inflammation and autophagy are closely linked to OA progression [7, 8]. Inflammation plays a pivotal role as a significant risk factor in the development of OA. The presence of the inflammatory cytokine interleukin (IL)-1β disrupts the anabolic processes of chondrocytes by suppressing collagen synthesis and oligoglycan production. Additionally, IL-1β stimulates the production of catabolic factors, including matrix metalloproteinases, as well as other inflammatory transmitters such as IL-6, prostaglandin E2, and nitric oxide (NO) [9–11]. Tumor necrosis factor (TNF)-α and IL-6 exert critically in OA progression [12, 13]. Autophagy occurs when cells phagocytose substances in their cytoplasm and transport them to lysosomes for degradation, meeting their metabolic needs, and renewing certain organelles [14]. Research has elucidated that the autophagy process in chondrocytes is diminished in both OA patients and mice models of OA. Subsequent to the suppression of autophagy in chondrocytes within animal models, AC undergoes a progressive deterioration and degenerative process. [15, 16]. Autophagy serves as a protective mechanism to sustain the normal functioning of cartilage, ensuring the maintenance of chondrocytes in a state of optimal health and the preservation of intracellular material and energy metabolism homeostasis. Consequently, the modulation of chondrocyte autophagy is hypothesized to potentially delay or modify the degenerative process of cartilage in OA [17, 18].

Saikosaponin D (SSD) is a triterpene saponin compound extracted from Radix Bupleuri with multiple pharmacological activities like anti-inflammatory [19], anti-oxidative stress [20], anti-tumor [21, 22], liver cell protection and liver fibrosis repression [23, 24]. Junsong Jiang et al. [25] found that SSD is a latent therapeutic drug for OA. SSD produces anti-inflammatory effects and can suppress nuclear factor κB (NF-κB) and mammalian target of rapamycin (mTOR) pathway to stimulate autophagy. There is speculation that SSD may delay OA progression through anti-inflammatory and autophagy modulation. Research was conducted to examine the pharmacological and molecular mechanisms of SSD therapy in OA to offer a clinical basis for treatment with SSD.

## Materials and methods

### Animals

Sixty healthy and clean male C57BL/6 mice (8–10 weeks of age and 20–23 g) were provided by Beijing Vital River Laboratory Animal Technology Co., Ltd. (Beijing, China). Mice were adaptively fed in a specific pathogen-free laboratory room with regular light exposure and free food and water for a week. All animal procedures were approved by the First Affiliated Hospital of Air Force Military Medical University Ethics Committee on Animal Experiments.

### OA model

A mouse OA model of mice was constructed [26]. Mice were anesthetized by intraperitoneal injection with 2% pentobarbital sodium (2 mL/kg), and the surgical area was routinely disinfected. Para-patella medial parapatellar incision in the right knee was performed, and skin and joint capsule were cut to expose the joint cavity. The patella was retracted laterally, and the knee joint was flexed as much as possible to expose the anterior cruciate ligament (ACL) and anterior angle of the medial meniscus. A cut was made at the anterior angle in order to remove the meniscus from the medial side, which was then resected. The amputation of the ACL was performed under direct view, and a front drawer examination confirmed that it had been completely severed. AC surfaces were protected during operation. The joint cavity was washed with normal saline and the joint capsule and skin were sutured layer by layer.

### Animal grouping and treatment

60 C57BL/6 male mice were divided into 6 groups (Sham group, OA group, 0 mg/kg SSD group, 0.5 mg/kg SSD group, 1.0 mg/kg SSD group, and SSD + miR-199-3p Antagomir group) with 10 animals in each group by random number table method. Sham group, joint cavity was exposed in the same way as the OA group, but the cruciate ligament and meniscus were not treated; OA group, OA modeling was performed on mice according to the above method; 0 mg/kg SSD group, mice were intragastrically administered with the same amount of Dimethyl Sulfoxide (DMSO; CS6719; Dingzhou Baikesaisi Biotechnology Co., Ltd, Dingzhou, China) after OA modeling; 0.5 mg/kg SSD group, mice were intragastrically administered with SSD (0.5 mg/kg/d) after OA modeling; 1.0 mg/kg SSD group, mice were intragastrically administered with SSD (1.0 mg/kg/d) after OA modeling; SSD + miR-199-3p Antagomir group, miR-199-3p Antagomir (GenScript, Nanjing, Jiangsu, China) was injected into the knee joint of mice 24 h before OA modeling, and mice were intragastrically administered with SSD (1.0 mg/kg/d) after OA modeling. SSD (purity ≥ 98%; S797046; Shanghai Macklin Biochemical Co., Ltd., Shanghai, China) was dissolved in DMSO.

### Tissue specimen treatment

All mice were euthanized eight weeks later. Briefly, mice were sacrificed with the nape facing up. Then, the front legs were immobilized and the skin and soft tissue were removed on the hind leg to make an incision at the knee joint. After exposing the tibial plateau, the surface resembling a regular translucent sphere (articular cartilage) was severed and processed. AC tissues were fixed with 4% paraformaldehyde, decalcified with 10% ethylene diamine tetraacetic acid, dehydrated with conventional gradient alcohol, and permeabilized with xylene. Paraffin-embedded sections were cut into 5 μm slices using a microtome (CUT4060, Leica, Germany). The sections were dewaxed and then subjected to hematoxylin–eosin (HE) staining, TdT-mediated dUTP-biotin nick end-labeling (TUNEL) staining, safranin O-fast green staining, and immunohistochemistry. AC tissues were obtained and preserved in liquid nitrogen for subsequent enzyme-linked immunosorbent assay (ELISA), reverse transcription quantitative polymerase chain reaction (RT-qPCR), and western blot.

### HE staining

HE staining was used to evaluate the histopathological condition of AC. Briefly, AC tissue sections were stained with hematoxylin (A600701-0010; Sangon Biotech, Shanghai, China), differentiated with 1% hydrochloric acid alcohol, and treated with 1% ammonia water. AC tissues were counter-stained with 1% Eosin solution (A600440-0025; Sangon Biotech, Shanghai, China). Next, AC tissue sections were dehydrated, cleared (75%, 90% and 95% ethanol, absolute ethanol, xylene × 2 times), dried, and sealed. The morphology and structure of AC were observed under an optical microscope.

### Safranin O-fast green staining

Safranin O staining was used to evaluate the damage of AC tissue. In short, AC tissue sections were stained with Weigert 's iron hematoxylin and then continuously incubated with 0.2% fast green solution (C500016-0500; Sangon Biotech, Shanghai, China), 1% ethylic acid solution, and 0.1% safranin O solution (A600815-0025; Sangon Biotech, Shanghai, China). Ultimately, the tissues were dehydrated, cleared, and mounted with neutral balsam. OA cartilage degeneration was assessed by 3 independent researchers according to the Osteoarthritis Research Society International (OARSI) grading method [27]. The grading ranges from Grade 0 (normal) to Grade 6 as intact cartilage and surface, Grade 0; intact surface, Grade 1; surface incontinuity, Grade 2; vertical fracture, Grade 3; erosion, Grade 4; denudation, Grade 5; and deformation, Grade 6.

### TUNEL staining

Chondrocyte apoptosis was detected by TUNEL method. In short, AC tissue sections were treated with 100 µl proteinase K (20 µg/ml; Roche, Shanghai, China) for 20 min at room temperature, and washed with 1 × PBS. Subsequently, chondrocyte apoptosis in the articular cartilage was measured using a cell death detection kit (11684817910; Roche), according to the manufacturer's protocols. Cells with brown nuclei were deemed TUNEL-positive and counted by a microscope in three fields of view/section. A light microscope (BX51; Olympus Corporation, Tokyo, Japan) was used to capture the images (magnification, ×200).

### Immunohistochemical staining

LC3-II in AC tissues was analyzed by immunohistochemistry. In short, AC tissue sections were treated with 3% H_2_O_2_ to remove endogenous peroxidase and blocked with 5% bovine serum albumin. Afterward, primary antibody LC3-II (1: 200; NB100-2220; Novus Biologicals, CO, USA) was added and then biotinylated Immunoglobulin G (IgG) (1:1000; ab6721; Abcam, Cambridge, UK) and streptavidin–peroxidase (SABC) (SA1027; Wuhan Boster Biological Technology Ltd., Wuhan, China) were incubated. Color development was done with DAB (AR1021; Wuhan Boster Biological Technology Ltd., Wuhan, China). Then, AC tissues were counter-stained with hematoxylin, differentiated with hydrochloric acid alcohol, and rinsed with warm water. Finally, the tissues were dehydrated with gradient alcohol, cleared with xylene, sealed with neutral gum, and observed under a microscope.

### Isolation and identification of chondrocytes

AC tissues were obtained from mice in the Sham and OA groups (one mouse from each group) and detached with 2% type II collagenase (LA10274; Biolab-Tech, Hangzhou, China). The detachment solution was centrifuged, and collected cells (about 1 × 10^6^ cells) were kept in Dulbecco’s Modified Eagle Medium (DMEM) (D930057; Shanghai Macklin Biochemical Co., Ltd., Shanghai, China) containing 10% fetal bovine serum (F917980; Shanghai Macklin Biochemical Co., Ltd., Shanghai, China). Then, cells were cultured in a 25 cm^2^ culture flask, and non-adherent cells were removed. Adherent cells were subcultured until primary cells were combined with slices. Articular chondrocytes at passages 1–3 were collected and identified by toluidine blue staining and type II collagen immunocytochemical staining.

Toluidine blue staining: Chondrocytes at passage 2 were fixed with 40 g/L neutral formaldehyde, stained with 1% toluidine blue (T992695; Shanghai Macklin Biochemical Co., Ltd., Shanghai, China), and observed under an optical microscope.

Immunocytochemical staining of type II collagen: Chondrocytes at passage 2 were fixed in 4% paraformaldehyde, treated with 50 μL 3% H_2_O_2_, and incubated with 50 μL Triton X-100 on ice. Then, chondrocytes were treated with 50 μL 5% blocking solution and combined with 50 μL primary antibody Col II polyclonal antibody (AB765P; 1:100; Millipore, MA, USA). PBS was taken as a negative control. Afterward, 50 μL goat anti-rabbit IgG secondary antibody (1:1000; ab6721; Abcam, Cambridge, UK) working solution was added, in combination with 50 μL SABC reagent. After incubation, color development was done with DAB, and chondrocytes were counter-stained with hematoxylin, differentiated with 0.5% hydrochloric acid, dehydrated with gradient ethanol, permeabilized with xylene, and sealed with neutral gum. Cytoplasm staining was observed under a light microscope. Type II collagen-positive staining cytoplasm was light yellow–yellow–brown.

### Cell grouping and transfection

In vitro, chondrocytes were divided into 9 groups. (1) Control group, normal chondrocytes isolated from the cartilage tissue of mice in the Sham group were not treated with SSD or transfected; (2) OA group, OA chondrocytes isolated from the cartilage tissue of the OA group mice were not treated with SSD or transfected; (3) 0 μmol/L SSD group, OA chondrocytes were treated with the same amount of 0.1% DMSO; (4) 5 μmol/L SSD group, OA chondrocytes were treated with 5 μmol/L SSD; (5) 10 μmol/L SSD group, OA chondrocytes were treated with 10 μmol/L SSD; (6) SSD (10 μmol/L) + miR-199-3p Inhibitor group: OA chondrocytes were treated with 10 μmol/L SSD and transfected with miR-199-3p Inhibitor (30 μm); (7) Mimic NC group: OA chondrocytes were transfected with mimic NC (30 μm) alone without SSD treatment; (8) miR-199-3p Mimic group: OA chondrocytes were transfected with miR-199-3p Mimic (30 μm) alone without SSD treatment; (9) miR-199-3p Mimic + pcDNA-TCF4 group: OA chondrocytes were transfected with miR-199-3p Mimic (30 μm) + pcDNA-TCF4 (30 μm) alone without SSD treatment.

miR-199-3p Inhibitor, miR-199-3p Mimic, Mimic NC, and pcDNA-TCF4 were synthesized by GenePharma (Shanghai, China). The above oligonucleotides or plasmid vectors were transfected into OA chondrocytes using Lipofectamine™ 2000 (11668030; Thermo Fisher Scientific, Waltham, MA, USA).

### 3-(4, 5-dimethylthiazol-2-yl)-2, 5-diphenyltetrazolium bromide (MTT) assay

Cell proliferation was detected by MTT assay. In short, a single cell suspension (2 × 10^5^ cells/mL) was seeded in a 96-well plate and cultured at 100 μL per well in serum-free DMEM. MTT (M6494; Thermo Fisher Scientific, Waltham, MA, USA) was added and incubated at 10 μL/well, and then chondrocytes were treated with Formazan dissolving solution at 100 μL/well. Absorbance at 570 nm (A_570_) was read on a microplate reader.

### Annexin V-fluorescein isothiocyanate (FITC)/propidium iodide (PI) staining

Apoptosis was detected by flow cytometry. In short, cells were detached with 0.25% trypsin (25200072; Thermo Fisher Scientific, Waltham, MA, USA) and centrifuged. According to the manufacturer's instructions, cells were treated with 5 μL Annexin V-FITC and 10 μL PI (V13242; Thermo Fisher Scientific, Waltham, MA, USA) and suspended in 400 μL 1 × Binding Buffer. FITC was tested in 30 min with a single emission laser with a wavelength of 480 mm. Data were analyzed by CellQuest software.

### ELISA

IL-1β, IL-6, and TNF-α in mouse cartilage tissue and chondrocyte supernatant were assessed in line with the procedures of the IL-1β (P10749), IL-6 (P08505), TNF-α (P06804) ELISA kits (Thermo Fisher Scientific, Waltham, MA, USA).

### RT-qPCR

Total RNA was collected with Trizol kit, and reverse transcription of complementary DNA (cDNA) was exerted using RNA reverse transcription kit (RR047A; Takara, Dalian, China). After PCR amplification, the product was verified by agarose gel electrophoresis. Ct was obtained by manually selecting the threshold at the lowest point of parallel rise of each logarithmic magnification curve. Data were analyzed by 2^−ΔΔCt^ method: ΔΔCt = [Ct (target gene)—Ct (reference gene)] the experiment—[Ct (target gene)—Ct (reference gene)] the control. PCR primers (Table 1) were designed and produced (Takara, Dalian, China).

**Table 1:** PCR primer sequence

Genes	Primer sequences
MiR-199-3p	F**:** 5’-GGCGGACAGTAGTCTGCAC-3′
	R**:** 5′-CCAGTGCAGGGTCCGAGG-3′
U6	F**:** 5′-CTCGCTTCGGCAGCACA-3′
	R**:** 5′-AACGCTTCACGAATTTGCGT-3′
TCF4	F**:** 5′-CCTGGCTATGCAGGAATGTT-3′
	R**:** 5′-CAGGAGGCGTACAGGAAGAG-3′
ATG1	F**:** 5′-GAGCTGCTTCACACTGAGGT-3′
	R**:** 5′-CCCAGCGAGATTCCCTCATC-3′
PI3K	F**:** 5′-AACACAGAAGACCAATACTC-3′
	R**:** 5′-TTCGCCATCTACCACTAC-3′
GAPDH	F**:** 5′-CTGGGCTACACTGAGCACC-3′
	R**:** 5′-AAGTGGTCGTTGAGGGCAATG-3′

### Western blot

Tissues and cells were added with radio-immunoprecipitation assay cell lysis buffer and protease inhibitor and lysed on ice. After that, proteins were collected after centrifugation and quantified using bicinchoninic acid kit. Then, 80 μL total protein was separated by 12% sulfate–polyacrylamide gel electro-pheresis and electroblotted onto a polyvinylidene fluoride membrane. After treatment with 5% skimmed milk powder, the membrane was incubated with primary antibodies TCF4 (1: 1000), Beclin1 (1: 1000), LC3 (1: 2000), Col2a1 (1: 1000), MMP-13 (1: 1000), glyceraldehyde-3-phosphate dehydrogenase (GAPDH) (1:20,000) (Abcam, Cambridge, UK) and with the goat anti-rabbit IgG or goat anti-mouse IgG (1: 2000; ab6721; Abcam, Cambridge, UK). Finally, chemiluminescence luminescence imaging was performed. Gray values were analyzed by Image Lab.

### The luciferase activity assay

Binding sites of miR-199-3p and TCF4 3′untranslated region (UTR) were predicted by the bioinformatics software https://cm.jefferson.edu/rna22. TCF4 3′UTR promoter region sequence containing miR-199-3p binding site was synthesized to construct the TCF4 3′UTR wild-type plasmid (TCF4-WT). Meanwhile, a TCF4 3′UTR mutant plasmid (TCF4-MUT) was constructed by mutating the binding site. TCF4-WT/MUT plasmids were transfected into mouse chondrocytes with Mimic NC and miR-199-3p Mimic, respectively. After 48 h, cells were lysed to determine luciferase activity using a dual luciferase reporter kit (E1910; Promega, Madison, WI, USA).

### Statistical analysis

Data analysis was performed with GraphPad Prism 8 (Graphpad, San Diego, CA, USA). Measurement data were represented in the form of mean ± standard deviation (SD). Two-group comparisons were done via t test. Comparisons among the multiple groups were done with one-way analysis of variance (ANOVA) and Tukey’s multiple comparisons test. *P* < 0.05 was accepted as indicative of significant differences.

## Results

### SSD alleviates cartilage damage in OA mice via elevating miR-199-3p

**Fig. 1:**
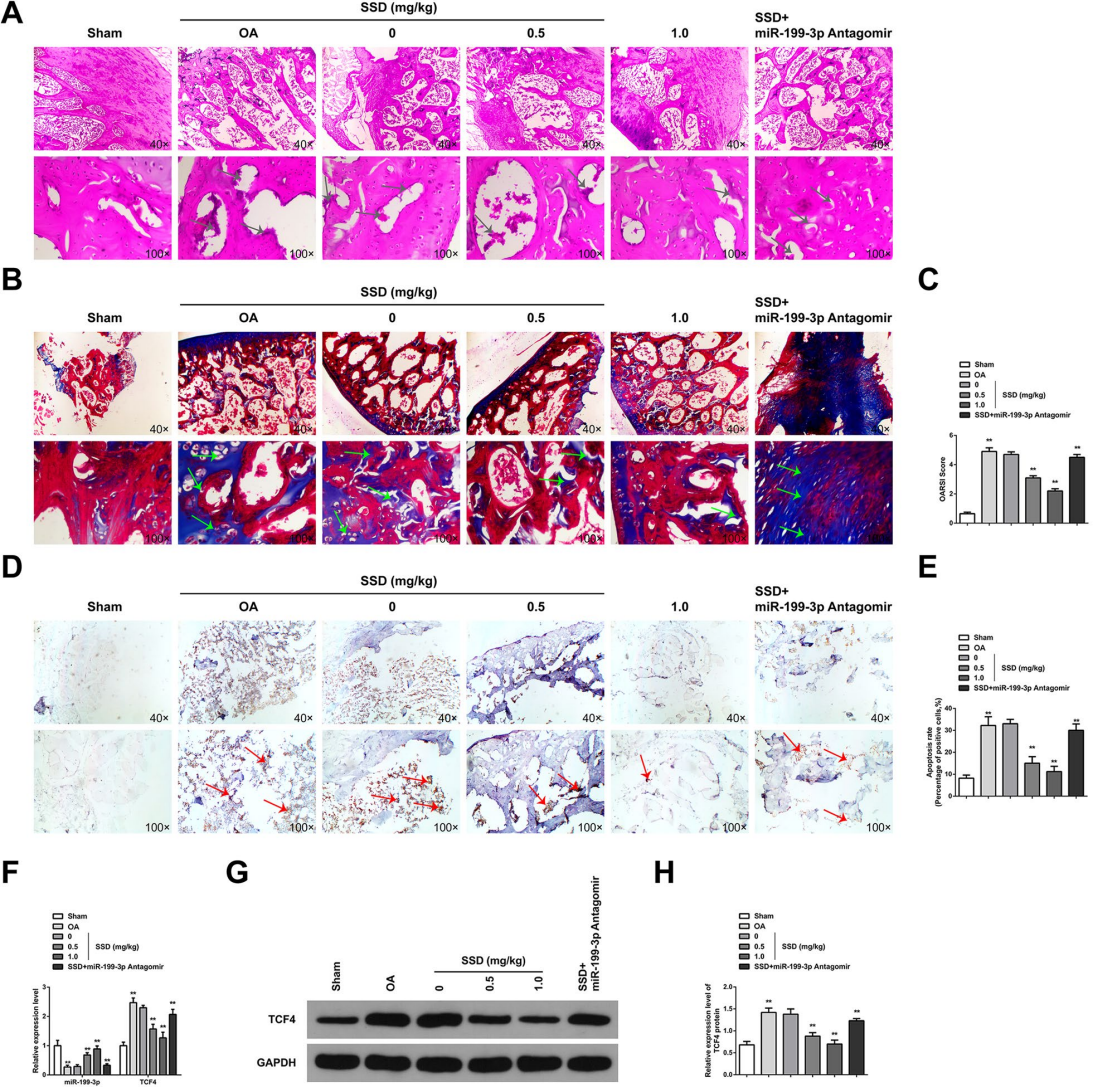
SSD relieves cartilage damage in OA mice via elevating miR-199-3p. **A** HE staining observed articular cartilage damage. The results showed that AC tissue in Sham group showed smooth surface and complete structure; while, OA cartilage tissue showed rough surface and cartilage destruction. SSD treatment improved cartilage damage, and downregulation of miR-199-3p weakened the protective effect of SSD on OA cartilage, arrows indicate cartilage damage; **B** Safranine O-fast green staining observed AC damage and showed that chondrocytes were regularly arranged in Sham group; while, the cartilage tissue degradation of OA mice was severe. SSD treatment could reduce the degradation of cartilage tissue, and downregulation of miR-199-3p weakened the protective effect of SSD on OA cartilage, arrows indicate cartilage degradation; **C** OARSI score evaluated the injury of AC, and the results showed that OARSI score increased in OA mice. SSD treatment could reduce OARSI score, and downregulation of miR-199-3p decreased the effect of SSD; **D**–**E** TUNEL staining tests showed that apoptosis of articular chondrocytes increased in OA mice, arrows indicate chondrocyte apoptosis. SSD treatment reduced the apoptosis of articular chondrocytes, and down-regulating miR-199-3p weakened the inhibitory effect of SSD on the apoptosis of OA chondrocytes; **F** RT-qPCR detected mRNA expression of miR-199-3p and TCF4. The results showed that miR-199-3p was downregulated and TCF4 mRNA was upregulated in the cartilage tissue of OA mice. SSD treatment promoted miR-199-3p expression and inhibited TCF4 mRNA, and downregulation of miR-199-3p decreased the effect of SSD; **G**–**H** Western blot detected TCF4 protein expression. TCF4 was upregulated in the cartilage tissue of OA mice. SSD treatment inhibited TCF4 protein expression, and downregulation of miR-199-3p decreased the effect of SSD. **A**,**B**,**D**, scale bar = 20 μm. **A**–**D**, the mice in each group. * *P* < 0.05, ** *P* < 0.01. n = 10

The mouse OA model was successfully constructed, and pathological changes in mouse cartilage tissue were observed (Fig. 1A–C). The surface of AC of mice in the Sham group was smooth and complete, the cartilage matrix was uniformly colored, the cartilage cell nucleus was blue-black or dark black, and the cell layer was clear and uniform in size. The cartilage structure of OA mice was seriously damaged, with cartilage fibrosis and cartilage thickening. The fissure extended deep to the thickened cartilage layer. The whole AC showed an irregular structure, and the OARSI score was significantly increased. Apoptosis detection was performed on cartilage tissues (Fig. 1D–E). The positive rate of apoptotic cells in OA mice was elevated. miR-199-3p was downregulated in OA mice; while, TCF4 was elevated (Fig. 1F–H). SSD treatment in OA mice alleviated pathological changes in cartilage tissues. SSD upregulated miR-199-3p in mouse cartilage tissues and suppressed TCF4 expressions. Silence of miR-199-3p partially mitigated SSD-mediated relief on cartilage injury in OA mice. In general, SSD alleviated cartilage damage in OA mice via upregulating miR-199-3p.

### SSD alleviates inflammation in OA mice and mediates autophagy via elevating miR-199-3p

**Fig. 2:**
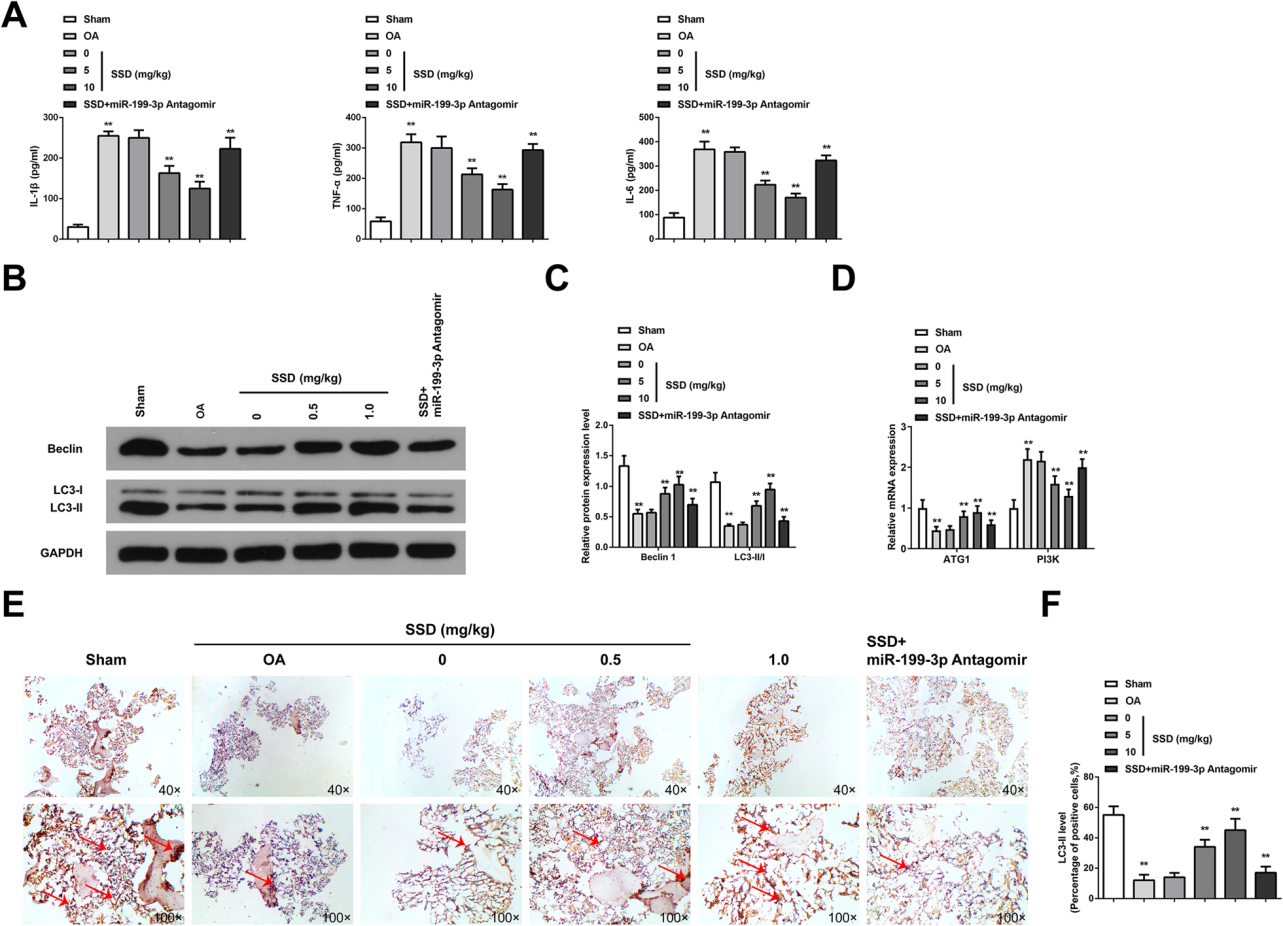
SSD relieves inflammation and mediates autophagy in OA mice via augmenting miR-199-3p. **A** ELISA measured content of pro-inflammatory cytokines IL-1β, TNF-α, and IL-6. The contents of IL-1β, TNF-α, and IL-6 in the cartilage tissues of OA mice were increased. SSD treatment reduced the contents of IL-1β, TNF-α and IL-6, and downregulation of miR-199-3p weakened the effect of SSD; **B**–**C** Western blot measured autophagy-associated protein Beclin1 and LC3-II/LC3-I ratio. Beclin1 and LC3-II/LC3-I ratio in OA mice cartilage decreased; while, SSD treatment increased Beclin1 and LC3-II/LC3-I ratio, and downregulation of miR-199-3p weakened the effect of SSD; **D** RT-qPCR detected autophagy-related genes (ATG1 and PI3K). ATG1 expression decreased and PI3K expression increased in OA mouse cartilage; while, SSD treatment increased ATG1 expression and decreased PI3K expression, and downregulation of miR-199-3p weakened the effect of SSD; **E**–**F** Immunohistochemical detected LC3-II protein showed that the positive expression of LC3-II decreased in the cartilage tissue of OA mice; while, SSD treatment increased the positive expression of LC3-II, arrow indicating positive expression of LC3-II. Downregulation of miR-199-3p weakened the effect of SSD. * *P* < 0.05, ** *P* < 0.01. n = 10

Inflammatory cytokines covering IL-1β, IL-6, and TNF-α have been verified to participate in OA development. Consequently, anti-inflammation is an impactive cure method to delay the onset of OA [28]. To investigate the effect of SSD on cartilage inflammation in OA mice, IL-1β, TNF-α, and IL-6 in mouse cartilage tissue were measured by ELISA. The results demonstrated that the content of inflammatory factors in OA mice was critically augmented, SSD reduced the content of IL-1β, TNF-α, and IL-6, while silencing miR-199-3p partially reversed the effect of SSD on the content of IL-1β, TNF-α, and IL-6 (Fig. 2A). It is indicated that SSD inhibits the inflammation of cartilage tissue in OA mice by regulating miR-199-3p. Autophagy-related proteins Beclin1 and LC3 were detected by Western blot. The results showed that Beclin1 and LC3-II/I ratio in cartilage tissue of OA mice decreased. SSD could promote Beclin1 expression and increase the ratio of LC3-II/I, while silencing miR-199-3p could partially reverse the effect of SSD on Beclin1 expression and the ratio of LC3-II/I (Fig. 2B, C). In addition, the mRNA expression of other autophagy-related genes (ATG1 and PI3K) was detected by RT-qPCR. The results showed that ATG1 mRNA expression decreased and PI3K mRNA increased in cartilage tissue of OA mice, and SSD could promote ATG1 mRNA expression and inhibit PI3KmRNA expression. Silencing of miR-199-3p could partially reverse the effects of SSD on ATG1 and PI3K mRNA expression (Fig. 2D). It is indicated that SSD promotes autophagy of chondrocytes in OA mice by regulating miR-199-3p. Meanwhile, the results of LC3-II immunohistochemical staining showed that the expression of LC3-II in cartilage tissue of OA mice was decreased, and SSD could promote the expression of LC3-II, while silencing miR-199-3p could partially reverse the effect of SSD on the expression of LC3-II (Fig. 2E, F). In conclusion, SSD alleviated inflammatory response and stimulated autophagy in OA mice via elevating miR-199-3p.

### Isolation and identification of chondrocytes

**Fig. 3:**
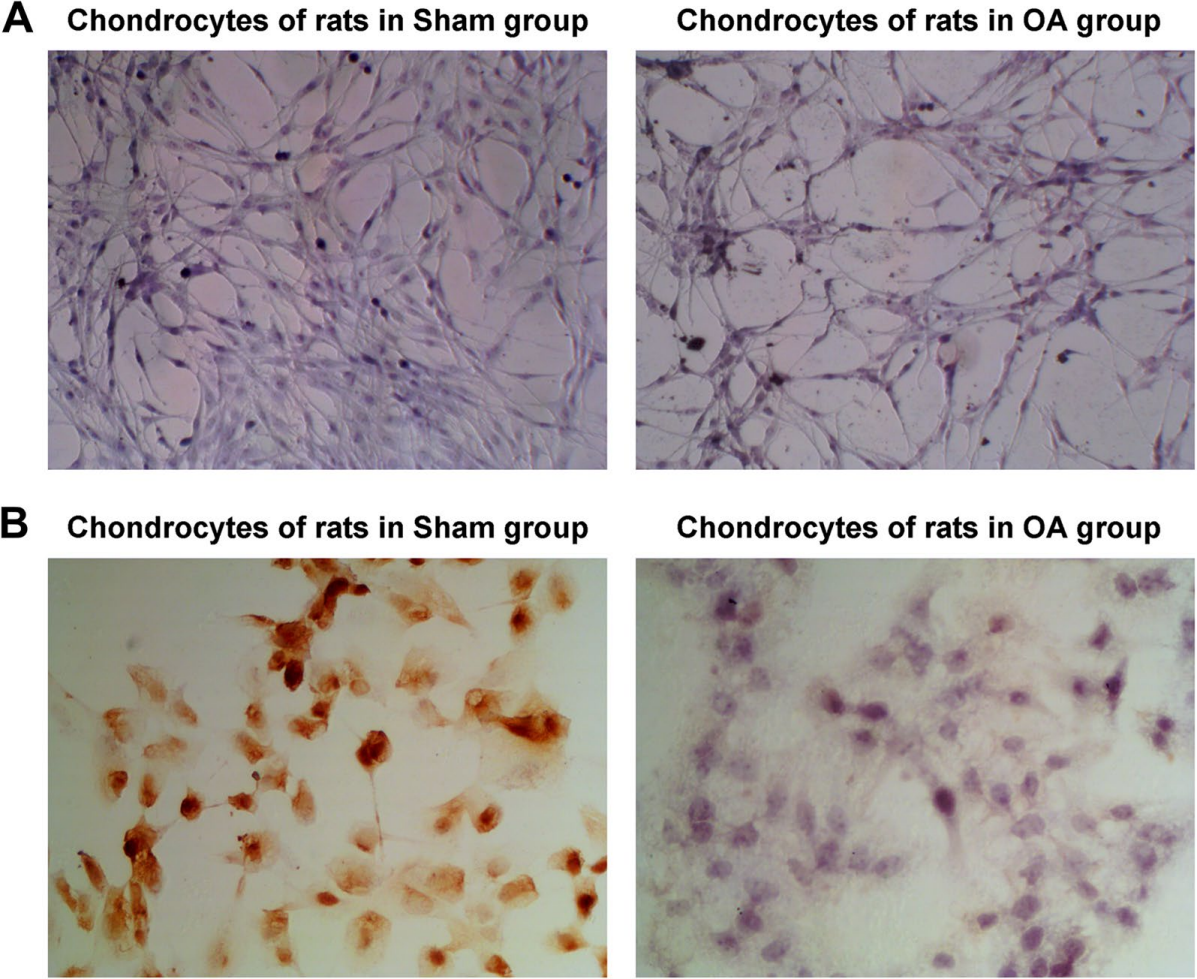
Isolation and identification of chondrocytes. **A** Toluidine blue staining identified chondrocytes. The chondrocytes isolated from rats in Sham group and OA group were blue and purple; while, the chondrocytes in OA group were reduced in number, sparsely arranged and with light staining; **B** Type II collagen staining observed the morphology of chondrocytes. COL2 was positive in the chondrocytes of rats in the Sham group, with brown particles visible in the cytoplasm, and the nucleus was basically unstained; COL2 staining was weak and positive in the chondrocytes of rats in the Model group, with a few light yellow particles in the cytoplasm, and the nucleus was basically unstained

Chondrocytes were identified. The results of toluidine blue staining showed that the chondrocytes isolated from the Sham group and OA group were blue-purple and identified as chondrocytes. In addition, the number of chondrocytes in the OA group was reduced, sparsely arranged, and lightly stained (Fig. 3A). The results of immunocytochemistry staining of type II collagen showed that COL2 staining of chondrocytes in Sham group was positive, brown granules were seen in the cytoplasm, and the nucleus was basically unstained. COL2 staining of chondrocytes in OA group was weak and positive, with a small amount of light yellow granules in the cytoplasm, and the nucleus was basically unstained (Fig. 3B).

### SSD alleviates inflammation of OA chondrocytes and mediates autophagy via elevating miR-199-3p

**Fig. 4:**
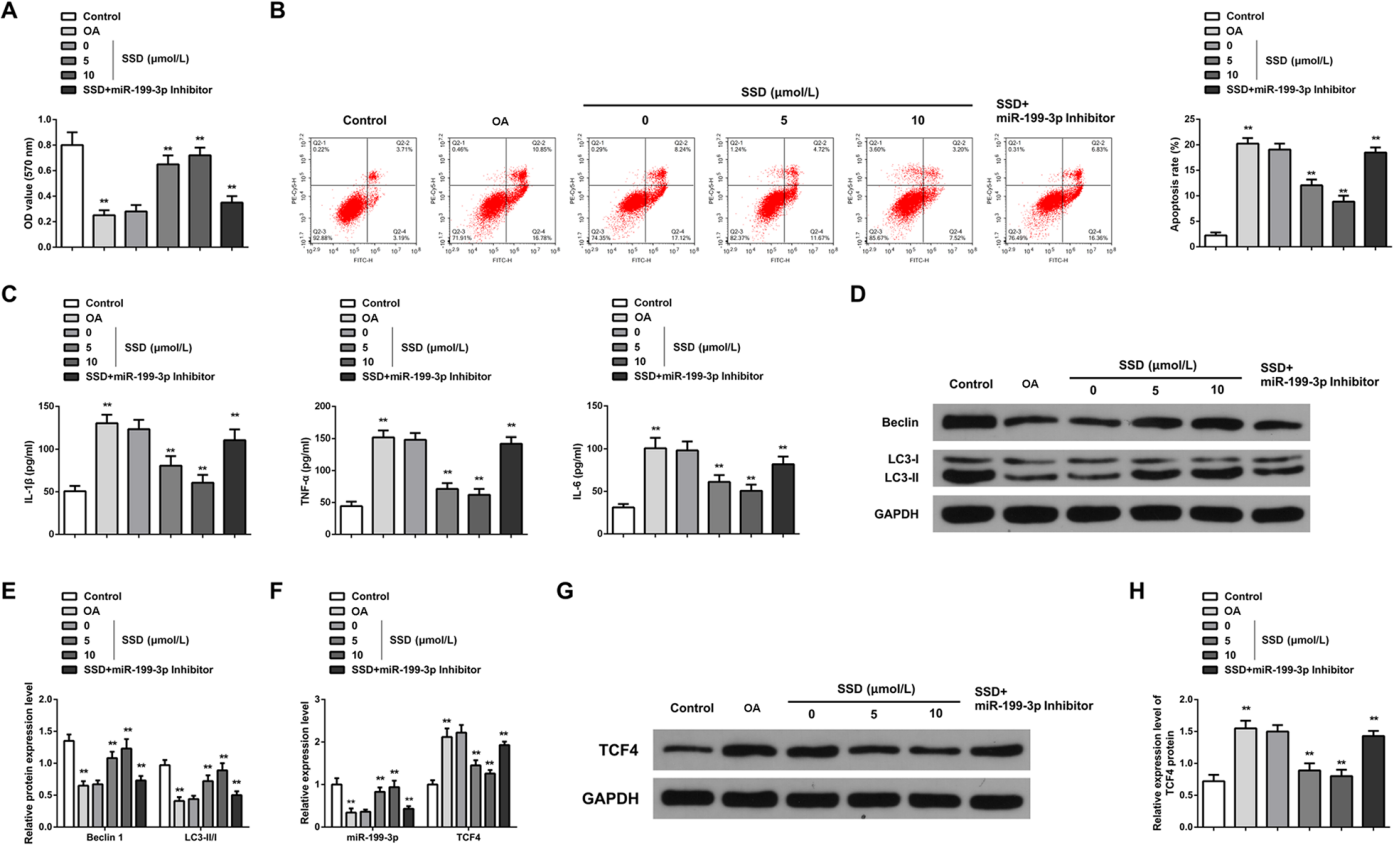
SSD relieves OA chondrocyte inflammation and controls autophagy via elevating miR-199-3p. **A** MTT analyzed cell proliferation. OA chondrocyte proliferation decreased; while, SSD treatment promoted cell proliferation. Downregulation of miR-199-3p attenuated the promoting effect of SSD on chondrocyte proliferation; **B** Flow cytometry measured cell apoptosis. OA chondrocyte apoptosis increased; while, SSD treatment inhibited apoptosis. Downregulation of miR-199-3p weakened the inhibitory effect of SSD on chondrocyte apoptosis; **C** ELISA measured content of pro-inflammatory cytokines IL-1β, TNF-α, IL-6 in the cell supernatant. The contents of IL-1β, TNF-α and IL-6 in the supernatant of OA chondrocytes were increased; while, the contents of IL-1β, TNF-α and IL-6 were decreased by SSD treatment. Downregulation of miR-199-3p weakened the effect of SSD; **D**–**E** Western blot detected autophagy-correlated Beclin1 and LC3-II/LC3-I ratio. Beclin1 and LC3-II/LC3-I ratio decreased in OA chondrocytes; while, SSD treatment increased Beclin1 and LC3-II/LC3-I ratio. Downregulation of miR-199-3p weakened the effect of SSD; **F** RT-qPCR evaluated miR-199-3p and TCF4 mRNA expression. The expression of miR-199-3p was downregulated and the expression of TCF4 mRNA was upregulated in OA chondrocytes. SSD treatment could promote the expression of miR-199-3p and inhibit the expression of TCF4 mRNA. Downregulation of miR-199-3p weakened the effect of SSD; **G**–**H** Western blot detected TCF4 protein expression. The expression of TCF4 protein was upregulated in OA chondrocytes; while, SSD treatment inhibited the expression of TCF4 protein. Downregulation of miR-199-3p weakened the effect of SSD. * *P* < 0.05, ** *P* < 0.01. N = 3

Chondrocyte viability, apoptosis, inflammation, and autophagy were measured (Fig. 4A–E). OA chondrocytes were characterized by low viability and autophagy and enhanced apoptosis and inflammation. After SSD cure, these changes were reversed; while, miR-199-3p Inhibitor mitigated the impact of SSD on OA chondrocytes. Col2a1 is a major cartilage extracellular matrix protein that is essential for normal cartilage function, and type II collagen-degrading MMP-13 contributes to cartilage degradation [29]. Western blot detection of Col2a1 and MMP-13 showed that Col2a1 protein expression decreased in OA chondrocytes; while, MMP-13 protein expression increased. SSD treatment could promote Col2a1 and inhibit MMP-13 protein expression. Downregulation of miR-199-3p could reduce the effect of SSD on Col2a1 and MMP-13 protein expression (Additional file 1: Fig. S1A, B). Additionally, miR-199-3p was downregulated in OA chondrocytes; while, TCF4 was upregulated. SSD treatment elevated miR-199-3p and suppressed TCF4 in OA chondrocytes (Fig. 4F–H).

### MiR-199-3p alleviates inflammation of OA chondrocytes and stimulates autophagy via targeting TCF4

**Fig. 5:**
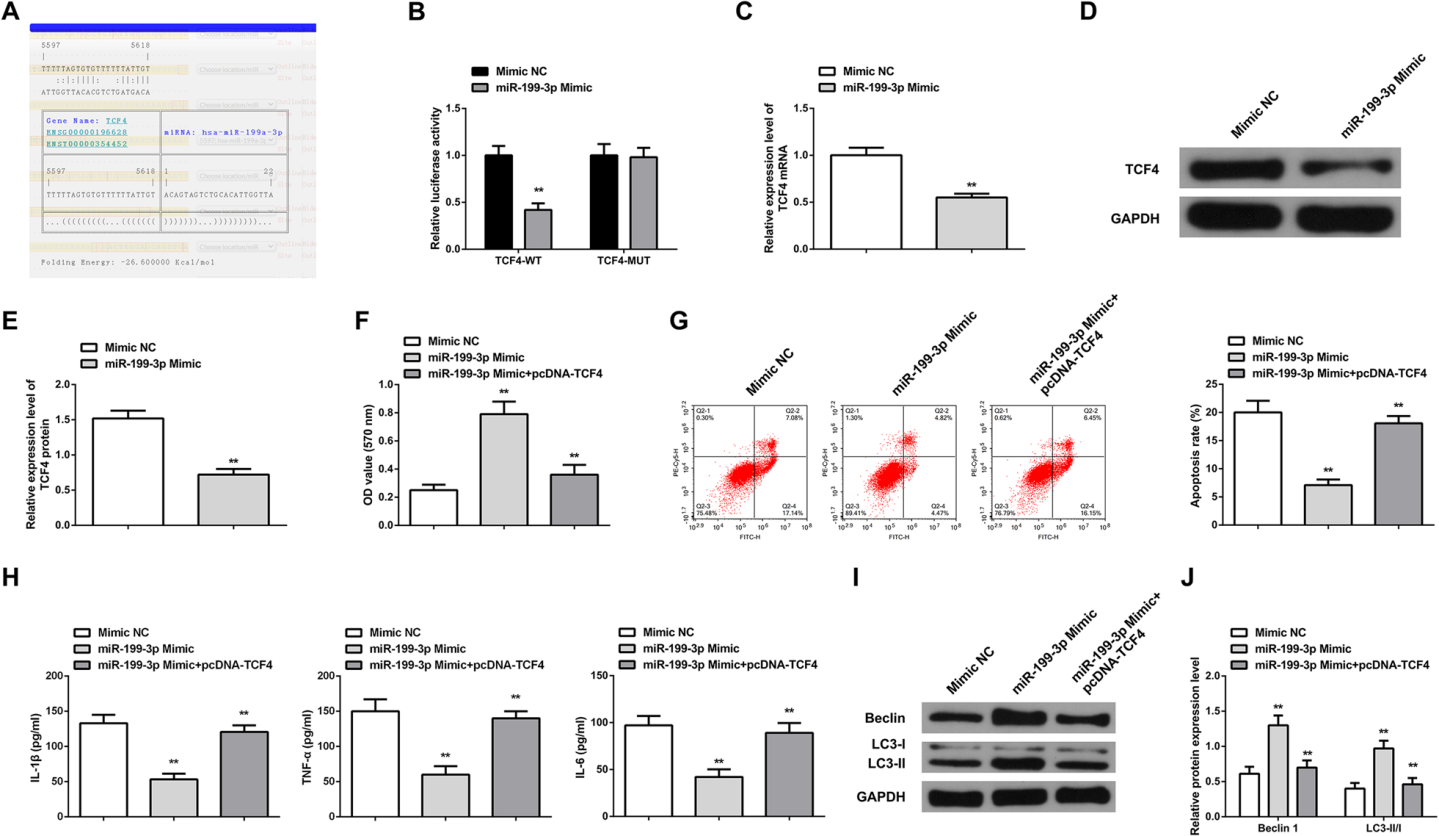
MiR-199-3p alleviates inflammatory response of OA chondrocytes and stimulates autophagy via targeting TCF4. **A** Bioinformatics website predicted the binding site between miR-199-3p and TCF4; **B** The luciferase activity assay verified the targeting relationship between miR-199-3p and TCF4. The results show that Chondrocytes co-transfected with TCF4-WT and miR-199-3p mimic showed decreased luciferase activity; **C**–**E** RT-qPCR and Western blot detected TCF4 mRNA and protein expression. After upregulation of miR-199-3p, the expression of TCF4 mRNA and protein decreased; **F** MTT measured cell proliferation. Upregulation of miR-199-3p promoted chondrocyte proliferation. However, upregulation of TCF4 can weaken the promoting effect of upregulation of miR-199-3p on cell proliferation; **G** Flow cytometry detected cell apoptosis. Upregulation of miR-199-3p inhibited cell apoptosis. Upregulation of TCF4 can weaken the inhibitory effect of upregulation of miR-199-3p on apoptosis; **H** ELISA detected contents of pro-inflammatory cytokines IL-1β, TNF-α, IL-6 in the supernatant. Upregulation of miR-199-3p reduced the contents of IL-1β, TNF-α, and IL-6. Upregulation of TCF4 can reduce the effect of upregulation of miR-199-3p; **I**–**J** Western blot measured autophagy-correlated Beclin1 and LC3-II/LC3-I ratio. Upregulation of miR-199-3p reduced Beclin1 and LC3-II/LC3-I ratios. Upregulation of TCF4 can reduce the effect of upregulation of miR-199-3p. * *P* < 0.05, ** *P* < 0.01. N = 3

The bioinformatics website manifested that miR-199-3p and TCF4 shared targeting sites (Fig. 5A). The targeting relationship between miR-199-3p and TCF4 was detected (Fig. 5B): Luciferase activity in chondrocytes co-transfected TCF4-WT and miR-199-3p Mimic was reduced.

TCF4 expression in OA chondrocytes was reduced after elevating miR-199-3p (Fig. 5C–E). miR-199-3p upregulation enhanced viability and autophagy while reduced apoptosis and inflammation in OA chondrocytes (Fig. 5F, J). Overexpressing TCF4 suppressed the influence of miR-199-3p upregulation on OA chondrocytes. In addition, after upregulation of miR-199-3p, Col2a1 protein expression was increased and MMP-13 protein expression was decreased; while, upregulation of TCF4 could reduce the effect of upregulation of miR-199-3p on Col2a1 and MMP-13 protein expression (Additional file 1: Fig. S1C, D).

## Discussion

OA is a degenerative condition influenced by various risk factors. Due to the incomplete understanding of the exact pathogenesis of OA, there is a dearth of effective pharmaceutical interventions and therapies for its management. Consequently, there is a pressing clinical need to investigate and formulate targeted pharmacological remedies for the treatment of OA. In this study, the beneficial therapeutic effects of bioactive SSD derived from Bupleuri on OA were analyzed.

SSD is the monomer with the strongest pharmacological activity extracted from saikosaponins extract. SSD can suppress multiple inflammatory processes. Chen Y et al*.* [30] indicated that SSD suppresses the content of IL-1β in carbon tetrachloride-stimulated hepatitis in mice. Likewise, Yao T et al*.* [31] stated that SSD reduced lipopolysaccharide-stimulated kidney injury by lessening the generation of pro-inflammatory cytokines in kidney tissue. SSD has also been verified to ameliorate lipopolysaccharide-stimulated inflammation-correlated depression-like behaviors via restraining neuroinflammatory response [32]. It is evident that SSD has a significant therapeutic effect and a wide range of applications in the anti-inflammatory field. Inflammation is a crucial risk factor for OA development, and inflammatory cytokines such as IL-1β, IL-6, and TNF-α have been verified to participate in OA. Therefore, anti-inflammation is a cure method to delay OA [28]. In this study, in *vivo* or in *vitro* experiments clarified that SSD effectively suppressed the content of pro-inflammatory factors IL-1β, IL-6, and TNF-α. The anti-inflammatory action of SSD might be ascribed to immediate suppression of these crucial inflammatory cytokines. A report has elucidated that SSD participates in the autophagic death of tumor cells via stimulating autophagosome formation [33, 34]. Wang B et al*.* [22] found that SSD accelerates autophagy via repressing the phosphorylation of mTOR signaling pathway. The mTOR signaling pathway is involved in chondrocyte autophagy. The critical pathological features of OA cartilage include the activation of mTOR pathway, repression of chondrocyte autophagy, reduced chondrocyte viability, elevated apoptosis, and lessened surviving chondrocyte quantities [35, 36]. Consequently, activating chondrocyte autophagy may be conducive to alleviating OA. In this study, SSD elevated autophagy proteins Beclin1 and LC3-II/LC3-I ratio and stimulated autophagy in chondrocytes to protect cartilage. In *vivo* and in *vitro* experiments also suggested that SSD effectively suppressed the apoptosis and inflammation of OA chondrocytes and elevated autophagy.

MiRNA, as a non-coding RNA molecule, is able to mediate cell development with inflammation and autophagy. Several studies have demonstrated the role of non-coding RNA in musculoskeletal diseases [37–39]. Multiple studies have elucidated that miRNAs are aberrant in OA tissues [40, 41]. The mechanism of action of SSD on OA was further discussed, and it was found that miR-199-3p was abnormal in OA and affected by SSD. Yu Chao et al*.* [42] maintained that miR-199 is silenced in synovia of patients with knee osteoarthritis. Fukuoka M et al*.* [43] stated that miR-199-3p is able to boost muscle regeneration and ameliorate aging muscles and muscular dystrophy. Gu W et al*.* [44] clarified that elevated miR-199-3p stimulates chondrocyte proliferation in KOA. miR-199-3p was downregulated in OA, while augmented miR-199-3p alleviated OA via restraining inflammation and controlling autophagy.

miRNAs frequently mediate target genes via combining with the UTR sequence of mRNA via a completely or incompletely complementary base paired mode, leading to limited translation or mRNA degradation [45, 46]. TCF4 was aberrantly expressed in OA and modulated by miR-199-3p. TCF4, as a critical risk gene on human chromosome 18, has been repeatedly reported to be elevated in OA [47] and participate in the occurrence and progression of OA [48–50]. Wang J et al. [51] maintained that TCF4 exerts a pro-inflammatory action via AMPK/NF-κB pathway. Nevertheless, silenced TCF4 is also deemed to stimulate autophagy [52]. The study manifested that TCF4 was augmented in OA. Elevated TCF4 was available to partially turn around the influence of miR-199-3p on OA, elucidating that miR-199-3p participated in the occurrence and progression of OA via targeting TCF4.

## Conclusions

In brief, SSD is available to alleviate inflammatory response of OA and stimulate autophagy via elevating miR-199-3p to target TCF4, thereby delaying OA development. The research offers a theoretical basis for SSD as a novel therapeutic drug for OA.

### Supplementary Information

**Additional file 1: Figure S1.** Effects of SSD and miR-199-3p on anabolic activity and catabolic activity of chondrocytes. A-D. Western blot detected Col2a1 and MMP-13. Col2a1 protein decreased and MMP-13 protein increased in OA chondrocytes. SSD treatment inhibited the expression of Col2a1 protein and promoted the expression of MMP-13 protein. Downregulation of miR-199-3p decreased the effect of SSD on the expression of Col2a1 and MMP-13 proteins. After upregulation of miR-199-3p alone, the expression of Col2a1 protein was increased, and the expression of MMP-13 protein was decreased; while, upregulation of TCF4 could reduce the influence of upregulation of miR-199-3p on the expression of Col2a1 and MMP-13 protein. * *P* < 0.05, ** *P* < 0.01. N = 3.

## Data Availability

The datasets used and/or analyzed during the present study are available from the corresponding author on reasonable request.
