# Supplementary figures and images for: Saikosaponin D alleviates inflammatory response of osteoarthritis and mediates autophagy via elevating microRNA-199-3p to target transcription Factor-4
Source: J Orthop Surg Res. 2024 Feb 22;19:151. doi: 10.1186/s13018-024-04607-0 (PMC10882832; doi:10.1186/s13018-024-04607-0)

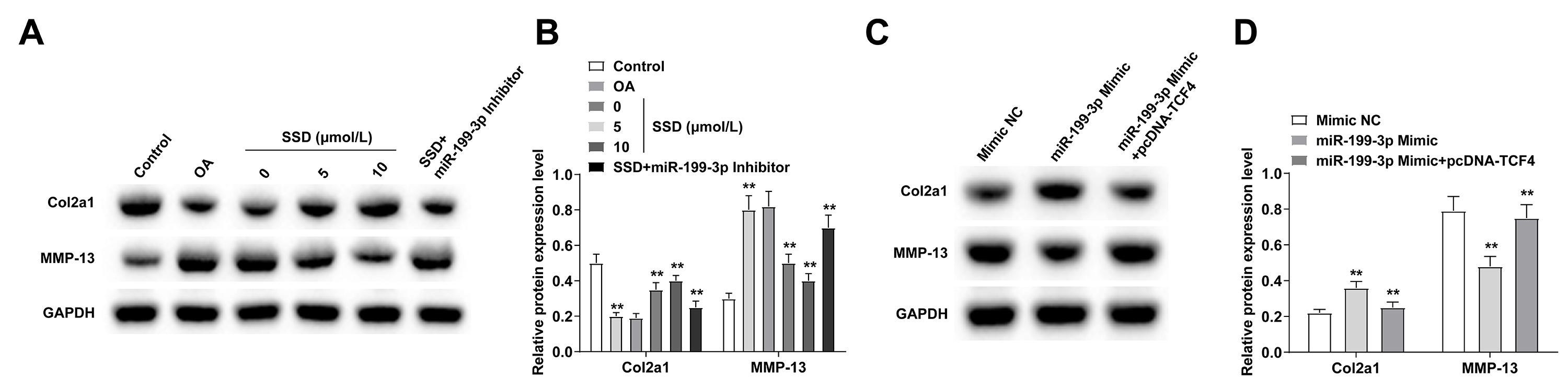

Supplement: Supplementary file 1 — Additional file 1: Figure S1. Effects of SSD and miR-199-3p on anabolic activity and catabolic activity of chondrocytes. A-D. Western blot detected Col2a1 and MMP-13. Col2a1 protein decreased and MMP-13 protein increased in OA chondrocytes. SSD treatment inhibited the expression of Col2a1 protein and promoted the expression of MMP-13 protein. Downregulation of miR-199-3p decreased the effect of SSD on the expression of Col2a1 and MMP-13 proteins. After upregulation of miR-199-3p alone, the expression of Col2a1 protein was increased, and the expression of MMP-13 protein was decreased; while, upregulation of TCF4 could reduce the influence of upregulation of miR-199-3p on the expression of Col2a1 and MMP-13 protein. * P < 0.05, ** P < 0.01. N = 3. [file 13018_2024_4607_MOESM1_ESM.tif]
